# Using the Age-Friendly Environment Framework to Assess Advance Care Planning Factors Among Older Adults With Limited Income: A Cross-Sectional, Descriptive Survey Study

**DOI:** 10.1093/geront/gnae059

**Published:** 2024-05-30

**Authors:** Christine Cleary Kimpel, Mary S Dietrich, Jana Lauderdale, David G Schlundt, Cathy A Maxwell

**Affiliations:** School of Nursing, Vanderbilt University, Nashville, Tennessee, USA; School of Nursing, Vanderbilt University, Nashville, Tennessee, USA; Department of Biostatistics, School of Medicine, Vanderbilt University, Nashville, Tennessee, USA; School of Nursing, Vanderbilt University, Nashville, Tennessee, USA; Department of Psychology, Vanderbilt University, Nashville, Tennessee, USA; School of Nursing, Vanderbilt University, Nashville, Tennessee, USA

**Keywords:** Aging, Future care planning, Perceived environment, Questionnaire, Socioeconomic status

## Abstract

**Background and Objectives:**

The World Health Organization created the Age-Friendly Environment (AFE) framework to design communities that support healthy aging and equitable decision making. This framework’s resource domains may account for disparately lower advance care planning (ACP) among older adults with limited incomes compared to those with high incomes. We aimed to describe and examine associations of AFE factors with ACP.

**Research Design and Methods:**

We recruited and conducted cross-sectional surveys among older adults with limited incomes in 7 community-based settings in Nashville, TN. ACP and AFE item scales were dichotomized and analyzed with unadjusted phi correlation coefficients.

**Results:**

Survey participants (*N* = 100) included 59 women, 70 Black/African American, and 70 ≥60 years old. Most participants agreed that their community was age friendly (≥58%) and varied in ACP participation (22%–67%). Participants who perceived easy travel and service access and sufficient social isolation outreach were more likely to have had family or doctor quality-of-life discussions (phi = 0.22–0.29, *p* < .05). Having a healthcare decision maker was positively associated with age-friendly travel, housing, and meet-up places (phi = 0.20–0.26, *p* < .05).

**Discussion and Implications:**

The AFE framework is useful for exploring the environmental factors of ACP, but further research is warranted to identify specific and immediate resources to support successful ACP among populations with socioeconomic disadvantage.

## Background and Objectives

Approximately 58 million older adults (aged 65+) live in the United States (17.3%; Caplan, 2023), and among these adults, the poverty rate has increased from 10.3% in 2021 to 10.9% in 2022 (Benson, 2023; Creamer et al., 2022). The long-term effects of socioeconomic disadvantage have the potential to lead to premature aging, multicomorbidity, and early mortality (Myers, 2009), necessitating early detection, intervention and management of illness, quality of life, and advance care planning (ACP) before reaching the age of 65.

Comprehensive ACP includes continuous learning, discussion, and documentation of care preferences with one’s trusted social ties (e.g., family, physicians) for future healthcare one may receive while unable to speak for oneself (Fried et al., 2009). Patients and healthcare decision makers (surrogates) participating in comprehensive ACP benefit from improved patient–provider communication, reduced decisional conflict, improved patient–surrogate alignment, and increased awareness of their disease trajectory (Malhotra et al., 2022). Unfortunately, older adults with low income are ~33% less likely to participate in ACP than those with higher income (Inoue, 2016; Nouri et al., 2020).

ACP factors among older adults with limited income include race, homelessness, number of comorbidities, mental health status, healthcare use, medical mistrust, and religious beliefs (Hong et al., 2018; Kaplan et al., 2020; Sudore et al., 2018). Although these factors are vital to understanding the complex picture of ACP inequities, these factors do not address the lack of community resources, which may contribute to this disparity. Recent research has described ACP disparities by neighborhood socioeconomic status, housing scores (e.g., housing size, rented vs owned), and housing communities (Barwise et al., 2019; Nouri et al., 2020; Tripken et al., 2018), but we found no published studies that investigated associations of the community environment (e.g., resources) with ACP.

Environment metrics include objective measures of the actual environment (e.g., number of hospitals) and subjective measures of the perceived environment (e.g., perceived neighborhood safety). The perceived environment entails the individuals’ cognitive and sensory experience, awareness, interpretation, and evaluation of the actual environment (Ma et al., 2014), partially contributing to health and healthcare planning behaviors (Marques et al., 2020). The World Health Organization’s (2015) Age-Friendly Environment (AFE) framework includes eight perceived physical and social environment domains of essential resources to support healthy and equitable aging (e.g., respect and social inclusion, proximity of services/resources). A research gap includes investigating whether perceived AFE factors influence ACP. Expanding this knowledgebase could inform future practice, policy, and research initiatives leading to improved ACP rates.

To address some of those gaps, we aimed to (a) describe age-friendly environmental factors and ACP behaviors and (b) examine associations of AFE factors with ACP behaviors among older adults with low income. Our hypotheses were as follows: (a) AFE scores (i.e., the level of age friendliness of the environment) and ACP behaviors would be low (75% of Stages of Change [SOC] scores will be ≤13.7; 75% of Age-Friendly Environment Assessment Tool (AFEAT) scores will be ≤28.8). (b) There would be a positive correlation of AFE factors with ACP readiness (i.e., *r* = 0.07–0.53).

## Research Design and Methods

### Design

A cross-sectional, descriptive survey design was used in Nashville, TN. The study protocol and procedures were approved by the institutional review board of a private university affiliated with an academic medical center in the southeastern part of the United States (IRB# 210905). This report follows the STROBE guidelines for reporting observational studies (Cuschieri, 2019). Supplementary Material are available that provide more detail about the conceptual framework, age eligibility criteria, and instruments.

### Participants and Setting

We used purposive and snowball sampling approaches to recruit participants. Inclusion criteria included the ability to self-report age ≥50, annual income <$20,000/year, independent living (i.e., not living in a nursing home), and to speak English. Exclusion criteria included significant hearing or vision loss that prevented participation. The age and income criteria were selected due to the premature aging risk in this population and to promote the inclusion of those in and near poverty ($12,880), respectively. Participants were screened for a stratification goal of 60% self-identifying as female, 70% of individuals self-identifying as Black or African American, and a roughly equal representation across broad age groups (50–59 years: 30%, 60–69: 40%, ≥70: 30%). These demographics were selected to promote sample heterogeneity based on key demographics by which ACP varies; older adults, women, and White Americans are more likely to participate in ACP than younger adults, men, and non-White Americans.

Given our stratified sampling approach, a sample of 100 was proposed to promote generalizable results for the population of interest and enable the detection of meaningful effect sizes for targeted future interventions. A sample of that size sufficed to detect phi coefficients as small as 0.33 (10% shared variance) with more than 80% statistical power.

From November 11, 2021, to June 9, 2022, we recruited participants from seven community-based settings frequented by older adults with low income in Nashville, TN. These seven sites included three affordable housing complexes, a community resource center, a recreation center senior program, a food pantry, and a church. None of these sites provided healthcare. Recruitment methods included posting physical flyers at recruitment sites and in-person flyer distribution. Individuals were screened by phone or in person regarding eligibility and stratification criteria. The enrolled participants (*n* = 100) were briefed on study procedures and scheduled for appointments. The nonprobability sampling mode included the risk of self-selection bias, which we mitigated by including seven recruitment sites, stratifying our sample, and offering incentives (Dillman et al., 2014).

### Instruments

The independent variables, AFE factors, were captured with the AFEAT, consisting of 10 items corresponding to the 8 social and physical AFE domains (e.g., respect and social inclusion, housing). Unlike objective measures of available resources (e.g., count of area grocery stores), this metric enabled participants to self-report agreement with perceptions of the environment on a 5-point Likert scale (1 = strongly disagree, 5 = strongly agree). Higher summed scores (10–50) and higher individual item responses corresponded with higher perceived age friendliness (Garner & Holland, 2019). The reliability of the total scores generated in this study was 0.81, slightly higher than a previously reported Cronbach’s alpha of 0.75 (Garner & Holland, 2019).

ACP behaviors, the dependent variables, were measured with the Stages of Change for ACP metric, a six-item instrument with each 5-point Likert-scale response option corresponding with one of the stages of change (e.g., 0 = precontemplation, 4 = maintenance) based on the Transtheoretical Model of Change (Fried et al., 2010). Each item corresponded with a core ACP behavior, including advance directive completion, identifying a healthcare decision maker (surrogate), and life-quality and life-sustaining measures discussions with family and doctors. Higher summed scores (0–24) and higher individual item responses (0–4) indicated higher levels of ACP. The reliability of the total scores generated in this study was 0.83.

The study questionnaire included items to assess ACP-related characteristics to describe the sample, including age, sex, race, ethnicity, marital status, employment, living arrangement, and education. We used the three-item Brief Health Literacy Screening (BHLS-3; possible scores: 3–15, higher score = higher health literacy; Wallston et al., 2014). The PROMIS 29 v2.1 four-item anxiety and four-item depression subscales were used. Raw scores were summed (4–20) and *T* scores were generated from the free online scoring service (healthmeasures.net) (Cella et al., 2010; Huang et al., 2019). We included a PRAPARE measure question to capture social contact (Weir et al., 2020). Functional frailty was assessed with the five-item FRAIL Scale (possible scores: 0–5, higher sum = higher likelihood of frailty; Woo et al., 2015). Religion and spirituality were measured with a nominal item of self-assessed spirituality level (Nair et al., 2020). Previous healthcare use in the last 2 years was evaluated with two questions from the Health and Retirement study (2020) questionnaire: any previous doctor visits (yes/no) and the number of hospitalizations.

### Data Collection

All instruments were included in a single questionnaire. Following electronic informed consent, the principal investigator administered the questionnaire in participant homes or in private recruitment site rooms by reading questions and recording responses using REDCap electronic data capture via a computerized tablet (Harris et al., 2009). REDCap (Research Electronic Data Capture) is a secure, web-based platform to support data management and integrity for research studies. Each participant received a $25 gift card incentive following survey completion. The mode of questionnaire administration included the risk of social desirability bias and acquiescence bias. We mitigated this risk by developing a trusting rapport with participants by facilitating approval from recruitment site leadership, treating individual participants as community experts, and emphasizing the importance of honest responses and the ability to refuse to answer any question (Dillman et al., 2014).

### Data Analysis

IBM SPSS Statistics 28. We used frequency distributions to summarize the nominal and ordinal categorical sample characteristics. Mean and standard deviation (*SD*) were used to summarize age, yet all the other continuous characteristic and study variable distributions were skewed; we used median, interquartile ranges (IQRs), and minimum and maximum values for those summaries. The fourth anxiety subscale item, “I felt uneasy,” was missing, thus no responses were available for analysis. Values for this missing item were pro-rated from the other item values. No other items were missing.

Likert responses to both the AFE and ACP measures were summarized using frequency distributions. Summaries of the overall scores for both of those measures used median and IQR.

Each variable was compared by age group (<60 vs ≥60). A chi-square test of independence was used for nominal variables and a Mann–Whitney test for ordinal and non-normally distributed continuous variables (e.g., AFEAT, ACP).

Given the bimodal or skewed item distributions of our independent and dependent variables, we dichotomized the distributions of the AFEAT and ACP item responses. The AFEAT “Strongly disagree,” “Disagree,” and “Neither agree or disagree” responses were combined into a single “No agree” category; the “Agree” and “Strongly Agree” responses were combined into an “Agree” category. The ACP scale item responses of “Action” and “Maintenance” responses were combined into a “Yes” category; “Precontemplation,” “Contemplation,” and “Planning” were combined into “No.”

After dichotomizing each item, we generated cross-tabulations of the AFEAT item responses with each ACP item response and assessed the strength of those associations using unadjusted phi coefficients, representing a correlation between two dichotomous variables. Interpretations of statistical significance were based on a maximum alpha of 0.05 (*p* < .05). The small sample size and individual item-response distributions prevented statistical adjustment.

## Results

### Sample Characteristics

Among those that were screened for eligibility or initial phone contact was attempted (*N* = 134), 34 were excluded: 5 for income (≥$20,000/year), 1 for age (<50 years), 5 declined, 18 were unreachable by phone, 2 because of the age stratification goal achievement, 2 for severe deafness/blindness, and 1 could not perform teach-back of the consent form. Participants’ sociodemographic and health characteristics are summarized for the overall sample and by age group (*N* = 100, Table 1). The mean age of the sample was 64.7 years (*SD* = 8.0). Although 59% (*n* = 59) self-reported as female, the group of participants that were ≥60 years had a higher proportion of females than the group <60 (70% vs 33.3%, *p* < .001). Seventy percent (*n* = 70) of the entire sample identified as Black or African American. Statistically significant differences were observed between age groups (<60 vs ≥60) for marital status and employment status (*p* < .05). Otherwise, no statistically significant differences between the age groups were observed (*p* > .05, Table 1).

**Table 1.** Sample Characteristics Overall and by Age Group (*N* = 100)

		Age groups					
	Overall (*N* = 100) *n* (%)	<60 (*N* = 30) *n* (%)	≥60 (*N* = 70) *n* (%)	Overall Median (IQR) min, max	<60 Median (IQR) min, max	≥60 Median (IQR) min, max	*p* Value
Sex (Female)	59 (59.0)	10 (33.3)	49 (70.0)				<.001
Race (Black/AA)	70 (70.0)	17 (56.7)	53 (75.7)				.075
Marital Status (Not married)	93 (93.0)	27 (90.0)	66 (94.3)				.023
Employment (Retired or on disability)	88 (88.0)	24 (80.0)	64 (91.5)				.008
Living situation (lives alone)	78 (78.0)	21 (70.0)	57 (81.4)				.206
Education (≤High school graduate)	54 (54.0)	19 (63.3)	35 (50.0)				.220
Spirituality or religiousness (Fairly or Very)	92 (92.0)	26 (86.6)	66 (94.2)				.640
Frequency of social contact (≥3 times a week)^a^	77 (79.3)	21 (75.0)	56 (81.2)				.581
Frailty Screening (frail or prefrail)	64 (64.0)	17 (56.7)	47 (67.1)				.398
Doctor visit in last 2 years (yes)	96 (96.0)	28 (93.3)	68 (97.1)				.373
No hospitalizations in the last 2 years (None)	46 (46.0)	14 (46.7)	32 (45.7)				.259
Health Literacy (BHLS-3) Possible scores: 3–15				13.0 (9.0, 14.8) 3, 15	12.5 (7.7, 15.0) 3, 15	13.0 (9.0, 14.0) 3, 15	.933
PROMIS 29 Anxiety				48.5 (40.3, 57.5) 40.3, 81.4	48.2 (40.3, 58.5) 40.3, 81.4	52.5 (40.3, 58.5) 40.3, 73	.975
PROMIS 29 Depression				48.9 (41.0, 58.7) 41.0, 79.3	49.1 (41.0, 60.0) 41.0, 79.3	48.9 (41.0, 58.3) 41.0, 79.3	.695
Age Friendliness (AFEAT) Possible scores: 10–50				39.0 (35.0, 43.0) 14, 50	39.5 (34.8,43.3) 22, 49	39.0 (34.8, 43.0) 14, 50	.690
ACP Behaviors (ACP SOC) Possible Scores: 0–24				12.0 (4.0, 16.0) 0.0, 24.0	11.5 (3.5, 16.0) 0.0, 24.0	12.0 (5.0, 18.3) 0, 24	.217

*Notes*: Age: *M* = 64.7, *SD* = 8.0, min = 50, max = 90. ACP = advance care planning; AFEAT = Age-Friendly Environment Assessment Tool; BHLS-3 = three-item Brief Health Literacy Screening; IQR = interquartile range; PROMIS = Patient-Reported Outcomes Measurement Information System; SD = standard deviation.

^a^ *N* = 97.

### Aim 1: Describe Age-Friendly Environment Factors

**Figure 1.**
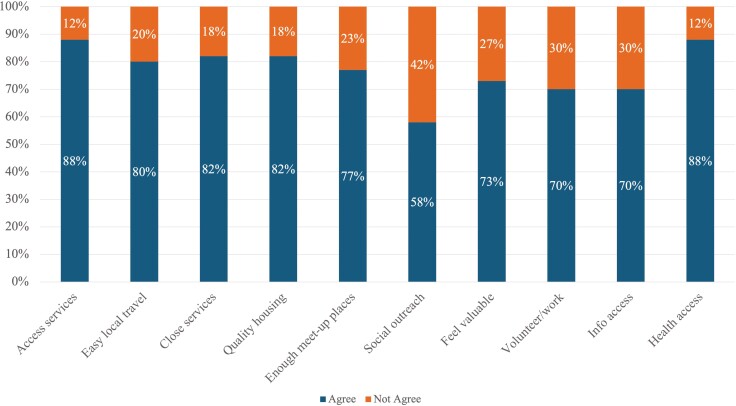
Age-friendly environment assessment tool dichotomized item distributions (%) (*N* = 100).

AFEAT overall scores tended to be high, with a median value of 39.0 out of a total possible score of 50 (IQR: 35.0, 43), indicating that most participants viewed their community environment as age friendly (see Table 1). No statistically significant difference in those scores was observed between the age groups (*p* = .690). Most participants agreed or strongly agreed with most of the survey items representing different parts of both the social and physical, community environment (≥70%; Items 1–5, 7, 9, 10, Figure 1). Sixty-two percent agreed or strongly agreed with the statement, “I can engage in volunteer or paid work without worrying about any special requirements I may have,” whereas only 58% of the participants agreed or strongly agreed with the statement, “There is consistent outreach to include people at risk of social isolation.”

### Aim 1: Describe Advance Care Planning Behaviors

**Figure 2.**
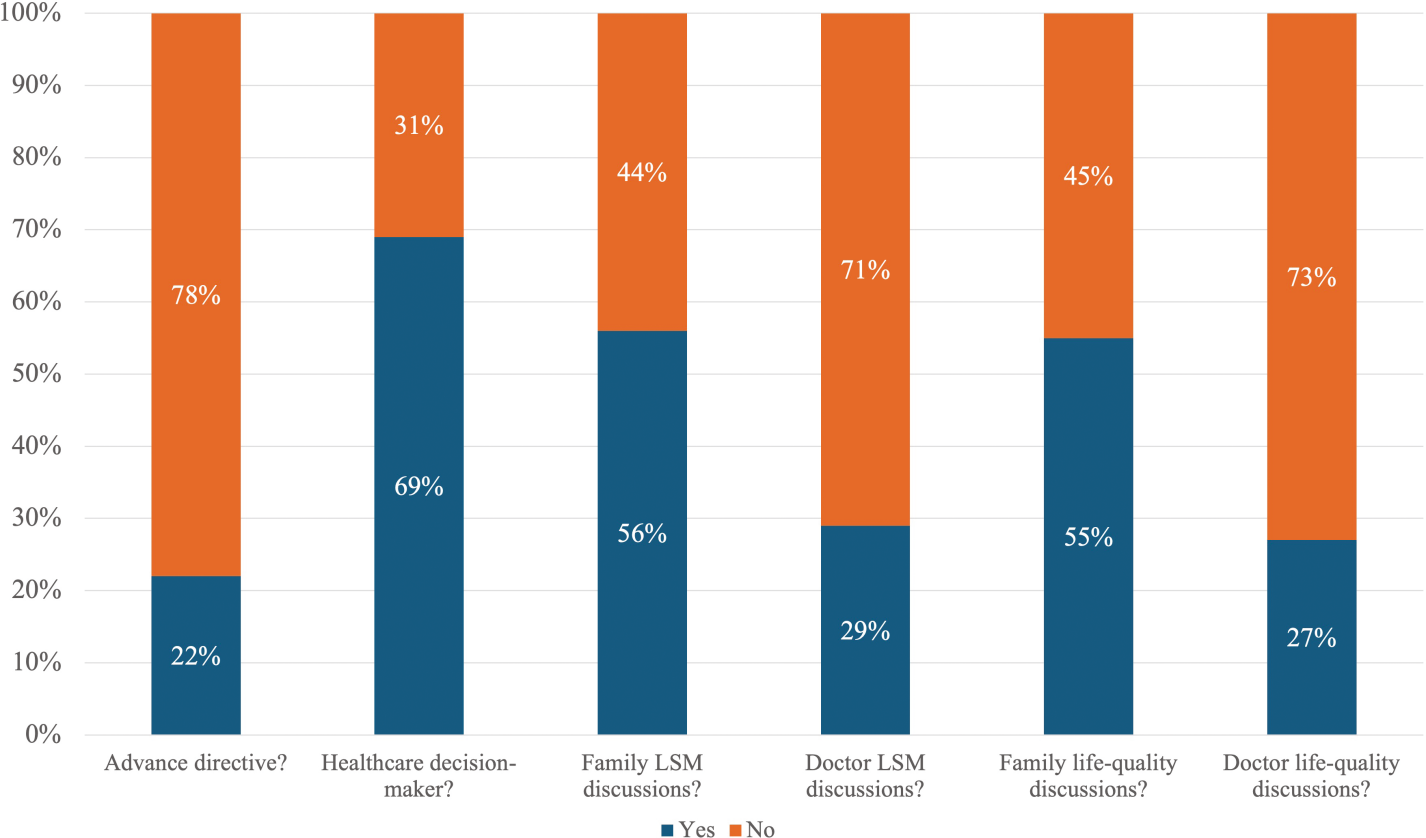
Advance care planning instrument dichotomized item distributions (%) (*N* = 100).

Median total scores for ACP behaviors were in the middle of the possible range from 0 to 24 (median = 12.0, IQR = 4.0, 16.0) and were not statistically significantly different between age groups (*p* = .217, Table 1). As depicted in Figure 2, only 22 (22%) of the participants reported having an advance directive, discussing life-sustaining measures with a doctor (29%), and discussing quality versus quantity of life with a doctor (27%). More than half of the participants reported having a healthcare decision maker (69%), family life-sustaining measures discussions (56%), and family life-quality discussions (55%).

### Aim 2: Associations of Age-Friendly Environment Factors With Advance Care Planning

Correlations of each individual AFE factor with each ACP behavior are reported in Table 2. Statistically significant associations of at least one AFE factor were observed with four of the six ACP behaviors: “Having a healthcare decision-maker,” “Life-Quality Family Discussions,” “Life-Sustaining Measures Family Discussions,” and “Life-Quality Doctor Discussions” (*p* < .05, Table 2).

**Table 2.** Correlational Matrix of Age-Friendly Factors With Advance Care Planning Behaviors

	Advance care planning behaviors (yes)					
Age-friendly Environment factors (agree)	Advance directive	Healthcare decision maker	Life-sustaining measures discussion with family	Life-sustaining measures discussion with doctor	Quality versus quantity discussion with family	Quality versus quantity discussion with doctor
(1) Easy access to local services	0.05	0.02	0.11	0.03	0.22*	0.23*
(2) Easy local travel	0.08	0.15	0.26**	0.04	0.25*	0.19
(3) Close proximity of local services	−0.07	0.14	0.18	−0.05	0.15	0.11
(4) Safe, clean, well-maintained housing	0.07	0.23*	0.14	0.03	0.13	0.07
(5) Plenty of meet-up places	0.12	0.20*	0.14	0.14	0.08	0.17
(6) Outreach for those at risk for social isolation	0.16	0.13	0.14	0.14	0.29**	0.24*
(7) Valuable community member	0.11	0.08	0.10	0.09	0.08	0.07
(8) Able to work or volunteer without restriction	0.07	−0.12	0.10	0.09	−0.00	0.15
(9) Access to community information and decision making	0.03	−0.06	0.08	0.08	0.13	0.15
(10) Easy access to health information and services	−0.10	−0.11	0.17	−0.04	0.16	0.02

*Notes*: Values in cells are phi coefficients; *p < .05; **p < .01.

#### Having a healthcare decision maker

Approximately 74.1% (60 of 81) participants who perceived age-friendly housing also reported having a healthcare decision maker whereas only 47.4% (9 of 19) of those who did not report having such an environment had a healthcare decision maker (phi = 0.23, *p* = .023). Of the participants that perceived sufficient meet-up places, 74.0 % (57 of 77) also reported having a healthcare decision maker whereas only 52.2% (12 of 23) that did not perceive this had a healthcare decision maker (phi = 0.20, *p* = .047).

##### Life-sustaining measures family discussion

A statistically significant positive association of easy local travel with life-sustaining measures family discussions (phi = 0.26, *p* = .009) but not with a doctor (phi = 0.04, *p* = .663). Of the participants who perceived easy local travel, 62.5% (50 of 80) also reported life-sustaining measures family discussions, whereas only 30.0% (6 of 20) of participants who did not perceive easy local travel also had such discussions (phi = 0.26, *p* = .009).

##### Life-quality family discussion

Positive associations of perceived easy access to local services, easy local travel, and social isolation outreach were observed with the life-quality family discussions (local services: phi = 0.22, *p* = .026; travel: phi = 0.25, *p* = .012; outreach: phi = 0.29, *p* = .004). Fifty-two of the 88 (59.1%) participants who perceived accessible local services also reported life-quality family discussions whereas only 25.0% (3 of 12) that did not report this perception also had such discussions. Similarly, 61.3% (49 of 80) participants who perceived easy local travel also had a life-quality family discussion, whereas only 30.0% (6 of 20) who did not perceive easy local travel also discussed this with family. Of the participants who perceived sufficient social isolation outreach, 67.2% (39 of 58) also had life-quality family discussions, whereas only 38.1% (16 of 42) who did not perceive enough social isolation outreach also had this type of discussion.

##### Life-quality doctor discussion

None of those (0 of 12) who reported not having access to local services also reported having a life-quality doctor discussion whereas 30.7% (27 of 88) of those who did perceive access had such a discussion (phi = 0.23, *p* = .025). Of those who perceived consistent social isolation outreach, 36.2% (21 of 58) also reported having a life-quality doctor discussion whereas only 14.3% (6 of 42) that did not perceive such outreach also had such discussions (phi = 0.24, *p* = .015).

## Discussion and Implications

### Key Results

To our knowledge, this study is among the first to use the AFE framework to explore the perceived environment with ACP among older adults with low income. Additionally, we stratified sampling by three demographic criteria (i.e., age groups, race, and gender) and recruited from seven community-based sites. Our key findings included that median AFE scores were higher than hypothesized, but the proportion of participants agreeing that the physical environment was age friendly was higher than the proportion agreeing that the social environment was age friendly. Median ACP scores were in the middle of possible scores, but individual items varied: more participants reported having a healthcare decision maker and family discussions than having an advance directive or doctor discussions. Finally, we observed eight positive and statistically significant associations of AFE factors with ACP behaviors among 60 unadjusted correlations. Among these findings, the strongest correlations were of easy local travel with life-sustaining measure family discussions and social isolation outreach with life-quality family discussions.

## Limitations

Aside from the previously mentioned associations, most associations observed in our study between AFE factors and ACP behaviors were very weak, and no associations were adjusted for potential confounders, given our limited sample size and dichotomized item responses. Although there may be, in fact, little to no correlation between AFE factors and ACP behaviors, the nature of our sample data distributions and sampling location may provide alternative explanations for the observed low correlations. In discussing our observed distributions of both environment perceptions and ACP behaviors, our study was conducted in a well-resourced location. Thus, while benefiting our participants, item distributions heavily skewed to one end or the other of a possible range of values and limited the potential for detecting correlations between the variables. The distributions were either so skewed or bimodal that we had to dichotomize them—making detection of correlations even more difficult. Correlations are best detected in samples with highly variable data representing the entire possible range of values and our sample did not meet those optimal criteria. Therefore, stronger correlations may exist between AFE factors and ACP behaviors yet could not be detected amid the limited value variability within our study sample.

Additionally, the possibility exists that AFE factors may have less influence on ACP behaviors than hypothesized when compared to the influence of variables that we did not measure or adjust for in the analysis. For instance, compared with AFE factors, knowledge, perceived norms, and decision-making preferences may have a stronger impact on ACP (Hong et al., 2018). Furthermore, specific ACP resources (lawyers, available documents) may have more of an influence on the likelihood of completing ACP behaviors than general community resources (i.e., AFE factors; Hong et al., 2018). Although we did not assess these other variables in this study, future research with a larger, more variable sample should identify salient resources for completing ACP actions.

Our sample stratification may have affected some of the observed statistical findings. For instance, our majority Black or African American-identifying sample may have less formal ACP (advance directives, physician discussions) due to medical mistrust from historical oppression and exploitation. Upon further statistical analysis, Black or African American identity was positively associated with identifying a healthcare decision maker (phi = 0.22, *p* = .027). Female identity was positively associated with identifying a healthcare decision maker (phi = 0.23, *p* = .020) and having life-sustaining measures family discussions (phi = 0.29, *p* = .004). Age was not statistically significantly associated with any ACP behaviors. Given these associations, future research with larger, more varied samples should statistically adjust for such confounders.

### Interpretation

Perceived AFE scores for this sample (median: 39.5, IQR 34.8, 43.3) were slightly lower compared with a previous sample of professional, well-educated participants (median: 44, IQR: 39, 47) (Garner & Holland, 2019), but still notably high. A previous AFE study that used self-reported and geographic data had similar findings of high perceived safety and sufficient social resources. However, the study’s geographic data yielded a more negative picture of the physical environment (e.g., insufficient bus stops and grocery stores) than was found in our self-report data (easy local travel and service access; Lehning et al., 2015). Survey responses pertaining to the perceived environment often differ from objective counts of community resources recorded by trained researchers (Marques et al., 2020). Future research should incorporate multiple measures to compare perceived resources with actual resources.

Environment perceptions are also influenced by each participant’s familiarity with available community resources (Blacksher & Lovasi, 2012; Sharkey, 2006). Our resource-savvy participants may differ from those in settings from which we did not recruit (healthcare). Additionally, participants may have differed in their selected environment—the setting of resources they envision when asked about their perceptions of the environment, which may have caused unintended variation in their AFE scores (Blacksher & Lovasi, 2012; Sharkey, 2006). Future research should statistically control for variation in each participant’s knowledge level of community resources and ascertain everyone’s selected environment.

Consistent with prior investigations, we observed low self-reported advance directive completion rates and low healthcare provider discussions among older adults with low income (Harrison et al., 2016; Ko et al., 2016). Lower rates of formal ACP may have been influenced by unmeasured variables such as ACP knowledge, medical mistrust, provider bias, and healthcare quality (Hong et al., 2018), which future research should capture and control for in the analysis.

Whereas our participants reported higher rates of having a healthcare decision maker than in previous studies, family discussion rates were consistent with the literature, suggesting that having a decision maker does not always lead to life-sustaining measures or life-quality conversations (Harrison et al., 2016; Ko et al., 2016). ACP preference discordance between patients and healthcare decision makers is common and occurs when patients assume their decision makers know their preferences without explicit conversation (Meghani & Hinds, 2015). This discordance increases decisional stress for caregivers during emergencies (Meghani & Hinds, 2015), and research should examine which social resources enhance meaningful discussion to reduce caregiver distress.

We observed the strongest positive and statistically significant associations of perceived easy local travel with life-sustaining measures family discussions (phi = 0.26) and of perceived social isolation outreach with life-quality family discussions (phi = 0.29). Robust community transit and social outreach may facilitate resilience, self-efficacy, healthcare access, and sociability (Levasseur et al., 2015), all of which may support ACP knowledge acquisition and discussion. Inadequate transportation access among older adults with low income, particularly those with disabilities, limits community participation (Bezyak et al., 2020) and delays healthcare intervention (Wolfe et al., 2020). Additionally, social isolation reduces ACP participation and community outreach is essential to prevent isolation in vulnerable populations (Cudjoe et al., 2019). Healthcare systems should assess patients for structural barriers (e.g., transportation) and build community partnerships to address such barriers.

Perceptions of safe, clean, and well-maintained housing and having sufficient meet-up places were also associated with having a healthcare decision maker. Perceived safety and adequate communal spaces encourage social interaction outside the home for older adults (Salvo et al., 2018), which is essential to support ACP, an inherently social activity. One solution to providing older adults with limited income with a more age-friendly setting is to provide permanent supportive housing—affordable housing with wraparound services, including ACP (Aubry et al., 2020; Pajka et al., 2023). Although most affordable housing falls short of this goal, individuals in these settings have more consistent access to resources than unhoused populations, providing the opportunity to connect with a support network and rebuild relationships with estranged family members (Kimpel et al., 2022; Padgett et al., 2020). Future research should continue to examine the influence of affordable housing on patient aging, social relationships, and ACP.

### Generalizability

These results generalize to similar populations in similar settings. The nonprobability sampling approach restricts generalizations to the broader socioeconomically disadvantaged population. Study participants exhibited near-average depression and anxiety scores, high health literacy scores, and higher socialization. Older adults with more complex social and mental health histories may have different environmental perceptions and ACP rates. Although this study sampled from seven sites and stratified by race, gender, and age, most sites were located within a highly resourced, metropolitan area. Future research should consider probability sampling in socioeconomically deprived, rural locations with varying resources.

## Implications and Conclusion

The WHO’s AFE initiative holds that community-led change is vital to prepare for the growth of our aging population. Healthcare systems and cities should collaborate to take a systematic and strategic approach to ACP implementation to prioritize policy change, inspire ACP awareness and acceptance across healthcare and community-based entities, and reduce patient and provider barriers (Ashana et al., 2022). Previous approaches to healthcare systems’ change include high-level leadership buy-in and commitment, a dedicated implementation team with healthcare and community representatives, team-based ACP delivery, and quality metrics for continuous evaluation and improvement (Bhatia et al., 2021). Community members should be surveyed to assess AFE factors to identify and address community priorities. Finally, public funding should support community health initiatives to provide home-based health visits and ACP as part of routine screenings for aging communities with socioeconomic challenges.

Several participants reported public health insurance coverage. Medicare reimburses healthcare providers for ACP conversations and this policy change has led to an increase in ACP conversation reimbursements for beneficiaries with an annual wellness visit (Palmer et al., 2021). However, Black, Hispanic, and dual-eligible beneficiaries were less likely to have an annual wellness visit than White or non-dual-eligible beneficiaries, representing an opportunity to address wellness visit disparities (Palmer et al., 2021). Additionally, ACP conversation reimbursements should include Medicaid-only billing and Medicaid should be expanded to all states with more flexible eligibility to increase opportunities for early and ongoing ACP.

In conclusion, research on the AFE framework with ACP is in its infancy, warranting studies that examine factors and influences within different community domains and levels. Our next steps include larger sampling and engaging community members and leaders. Additionally, we plan to explore associations of psychosocial coping, resilience, and childhood experiences with ACP in an affordable housing setting to create an AFE for adults with limited income.

## Supplementary Material

gnae059_suppl_Supplementary_Materials

## Data Availability

We will make our data available to other researchers, particularly those in the early stages of their careers at our institution. We will share our analytic methods and data collection materials to researchers within and beyond our institution upon email request and with appropriate institutional permissions. For those inside the institution, data can be accessed upon request in REDCap. This study was not pre-registered with any independent institutional registry.
