# Supplementary material for: Using the Age-Friendly Environment Framework to Assess Advance Care Planning Factors Among Older Adults With Limited Income: A Cross-Sectional, Descriptive Survey Study
Source: Gerontologist. 2024 May 30;64(7):gnae059. doi: 10.1093/geront/gnae059 (PMC11192857; doi:10.1093/geront/gnae059)
Supplement: gnae059_suppl_Supplementary_Materials [file gnae059_suppl_supplementary_materials.docx]

Supplementary Material

Conceptual Framework Rationale

The rationale for this study drew on two theoretical models: the Transtheoretical Model of Change and the Ecological Model for Active Living (Prochaska and Diclemente 1982; Sallis et al., 2006). The Transtheoretical Model holds that behavior change occurs in a series of stages, beginning with Pre-contemplation and continuing to relapse or maintenance of the desired behavior change. This theory has been used extensively with psychological interventions (e.g., smoking cessation) and is a foundational theoretical framework that underpins several advance care planning (ACP) metrics and studies. The focus of this theory includes using processes of change (e.g., consciousness-raising) to change behavior, which is highly useful for individual-level behavior change; however, given the national disparity between socioeconomic levels, we looked beyond this theory to consider contextual factors that may influence differences in ACP behavior change among older adults with limited income. Then we considered the Ecological Model of Active Living, which provides a multi-level (e.g., individual, behavior setting, community, etc.) and multi-dimensional (e.g., physical, policy, and social dimensions) framework for multi-level interventions to increase physical activity. Supplementary Figure 1 highlights the combined framework and how we operationalized the variable.

Supplementary Figure 1. Framework for conceptual and operational variables


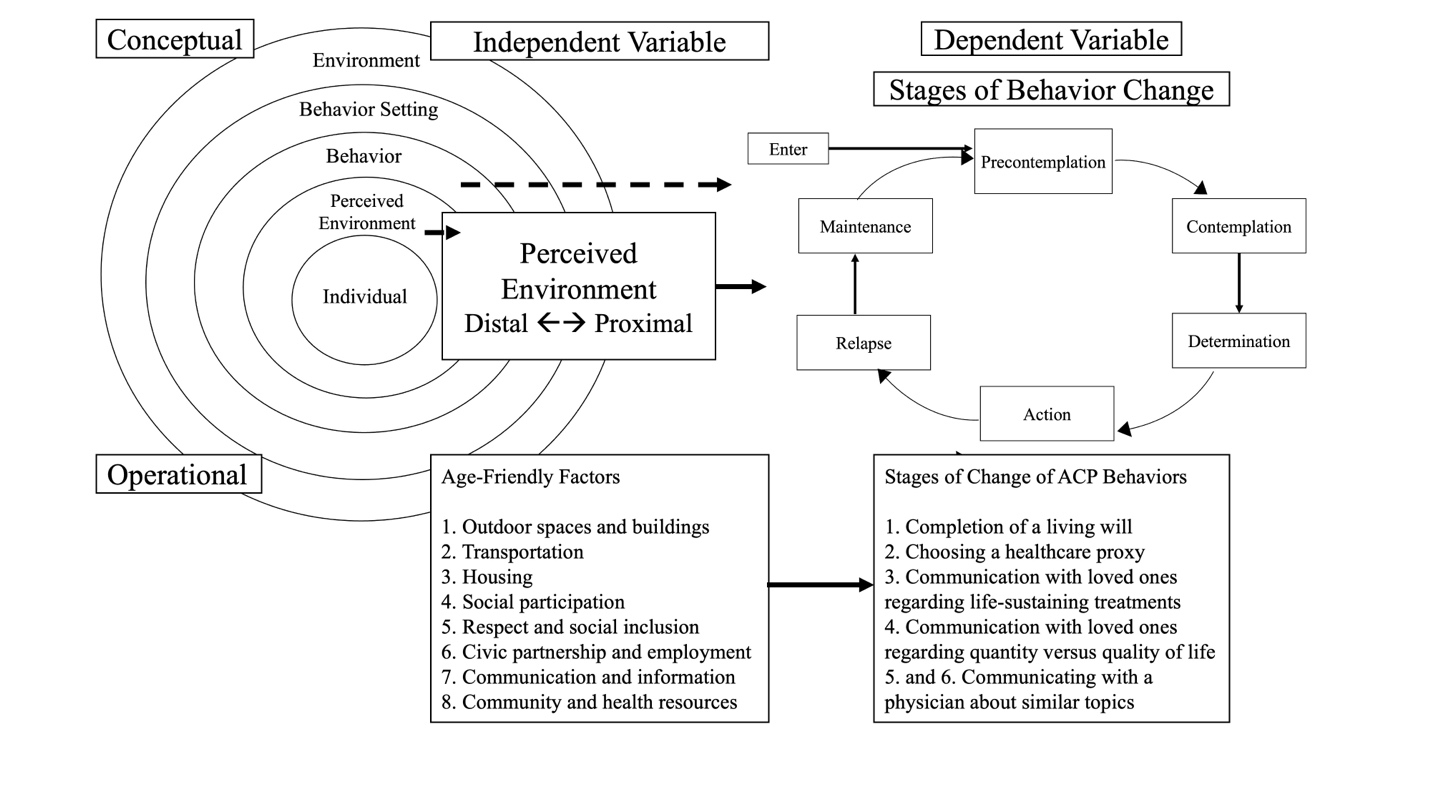


This figure depicts the conceptual level of the independent variable, informed by the Ecological Model of Active Living, and of the dependent variable, derived from the Stages of Change Model, which is part of the Transtheoretical Model of Change. The independent variable, the perceived environment, is operationalized as the Age-Friendly Environment factors. The dependent variable, the stages of behavior change, is operationalized as the Stages of Change of advance care planning behaviors.

The rationale for enrolling individuals aged 50 to 59 years old

Adults with limited income may experience chronic low income and other social, economic, and material disadvantages. Additional exposure to chronic stress likely produces harmful effects on the body that may lead to premature aging and illness onset. Based on previous findings, we stratified our sample to include individuals aged 50 to 59 years to capture survey responses among those who may not be in the chronologically older age category but may have experienced advanced biological aging.

Detailed Instrument Description

The study questionnaire included items to assess ACP-related characteristics to describe the sample and for statistical adjustment. Health literacy, anxiety, and depression were assessed because limited health literacy and higher rates of psychological issues may affect the ability to participate fully in ACP (Kelly et al., 2021; McMahan et al., 2020; Nouri et al., 2019). The Brief Health Literacy Screening (BHLS-3) is a 3-item measure with higher summed scores (3-15) indicating greater confidence and independence in reading and comprehending healthcare materials. The reliability of our scores was 0.81, consistent with prior studies (⍺=0.76, 0.80) (Wallston et al., 2014). The PROMIS 29 v2.1 4-item anxiety and 4-item depression subscales were used. Raw scores were summed (4-20) from 5-point Likert scale items (1=never, 5=always), and T scores were generated from the free online scoring service (healthmeasures.net) (Cella et al., 2010). The reliability of our scores were 0.90 for the anxiety subscale and 0.93 for the depression subscale, corresponding to previously reported scores of 0.87 (anxiety) and 0.89 (depression) (Huang et al., 2019).

Sample characteristics also included age at the time of the interview, sex, race, ethnicity, marital status, employment, living arrangement, and education. A single social determinant of health, social contact, was extracted from the PRAPARE measure and adapted to the survey (Weir et al., 2020). Functional frailty was assessed with the 5-item FRAIL Scale (e.g., are you able to climb a flight of stairs), providing a summed count (0-5) of yes responses (1=yes) to screen for the likelihood of frailty with a higher count (Woo et al., 2015).

Religion and spirituality were measured with a nominal item of one’s self-assessed level of spirituality (Nair et al., 2020). Previous healthcare use in the last two years was evaluated with two questions from the Health and Retirement study (2020) questionnaire: any previous doctor visits (yes/no) and the number of hospitalizations.

References

Kelly, E.P., Henderson, B., Hyer, M., & Pawlik, T.M. (2021). Intrapersonal factors impact advance care planning among Cancer patients. *American Journal of Hospice and Palliative Medicine, 38*(8), 907-913. http://doi.org/10.1177/10499091209624

McMahan, R.D., Barnes, D.E., Ritchie, C.S., Jin, C., Shi, Y., David, D., Walker, E.J., Tang, V.L., & Sudore, R.L. (2020). Anxious, depressed, and planning for the future: Advance care planning in diverse older adults. Journal of the American Geriatrics Society, 68(11), 2638-2642. http://doi.org/10.1111/jgs.16754

Prochaska, J.O. & Diclemente, C.C. (1982). Transtheoretical therapy: Toward a more integrative

model of change. *Psychotherapy: Theory, Research & Practice*, 1982. *19*(3): p. 276-288.

https://doi.org/10.1037/h0088437

Sallis, J. F., Cervero, R. B., Ascher, W., Henderson, K. A., Kraft, M. K., & Kerr, J. (2006). An

ecological approach to creating active living communities. *Annual review of public*

*health*, *27*, 297–322. https://doi.org/10.1146/annurev.publhealth.27.021405.102100
